# Fever in Liverpool

**Published:** 1847-07

**Authors:** 


*Fever in Liverpool.*—We regret to state that there has been an alarming increase of fever and mortality in Liverpool during the last week, and that the disorder is not by any means confined to the lower classes. Two Roman Catholic clergymen have fallen victims to the pestilence. In addition to the deaths of Mr. Parker and Dr. Kelley, we have to report that Inspector Forsyth, one of the recently-appointed relieving-officers, after an illness of eight days, died on Friday morning. Mr. Gray, one of the overseers, is seriously indisposed. The same may be said of Mr. Staine and Mr. Lamonby, two of the relieving-officers, and also of three of the medical gentlemen connected with the parish, namely, Mr. Steele (only a few days appointed to the office,) Dr. Robert Gee, and Mr. Grimsdale, surgeon to the work-house. Three of the policemen employed as district relieving-officers have become afflicted with typhus; and an able-bodied pauper from the work-house, who was employed as an assistant in the new parish offices in St. Anne Street, caught the same malignant disease at those offices, and is dead.—*Ibid.*

